# Clinical application of patient-specific 3D printing brain tumor model production system for neurosurgery

**DOI:** 10.1038/s41598-021-86546-y

**Published:** 2021-03-26

**Authors:** Yun-Sik Dho, Doohee Lee, Teahyun Ha, So Young Ji, Kyung Min Kim, Ho Kang, Min-Sung Kim, Jin Wook Kim, Won-Sang Cho, Yong Hwy Kim, Young Gyu Kim, Sang Joon Park, Chul-Kee Park

**Affiliations:** 1 Department of Neurosurgery, Chungbuk National University Hospital, Chungbuk National University College of Medicine, Cheongju, Republic of Korea; 2 MEDICALIP Co. Ltd., Changgyeong Building, 174, Yulgok-ro, Jongno-gu, Seoul, 03127 Republic of Korea; 3 Department of Neurosurgery, Seoul National University Bundang Hospital, Seongnam, Republic of Korea; 4 Department of Neurosurgery, Seoul National University Hospital, Seoul National University College of Medicine, Daehak-ro 101, Jongno-gu, Seoul, 03080 Republic of Korea; 5 Department of Radiology, Seoul National University Hospital, Daehak-ro 101, Jongno-gu, Seoul, 03080 Republic of Korea

**Keywords:** CNS cancer, Brain, Surgical oncology, CNS cancer, Brain imaging, Three-dimensional imaging, Surgery

## Abstract

The usefulness of 3-dimensional (3D)-printed disease models has been recognized in various medical fields. This study aims to introduce a production platform for patient-specific 3D-printed brain tumor model in clinical practice and evaluate its effectiveness. A full-cycle platform was created for the clinical application of a 3D-printed brain tumor model (3D-printed model) production system. Essential elements included automated segmentation software, cloud-based interactive communication tools, customized brain models with exquisite expression of brain anatomy in transparent material, adjunctive devices for surgical simulation, and swift process cycles to meet practical needs. A simulated clinical usefulness validation was conducted in which neurosurgeons assessed the usefulness of the 3D-printed models in 10 cases. We successfully produced clinically applicable patient-specific models within 4 days using the established platform. The simulated clinical usefulness validation results revealed the significant superiority of the 3D-printed models in surgical planning regarding surgical posture (*p* = 0.0147) and craniotomy design (*p* = 0.0072) compared to conventional magnetic resonance images. The benefit was more noticeable for neurosurgeons with less experience. We established a 3D-printed brain tumor model production system that is ready to use in daily clinical practice for neurosurgery.

## Introduction

Since 3-dimensional (3D) printing was introduced to the scientific field approximately 25 years ago, remarkable advances and recent generalizations in technology have enabled 3D printing-related medical devices to be rapidly applied in many medical fields, proving their necessity^1–4^. One of the most promising areas for the clinical application of 3D printing technology is surgical simulation systems for anatomical disease models. Patient-specific disease models, guaranteeing precise definition of disease extent and detailed reflection of related anatomical structures, are undoubtedly useful in planning surgery for individual patients^5–7^. To apply this patient-specific surgical planning system to real-world clinical practice, a well-organized interactive process ranging from design to production in a reasonable time is a vital element. In the field of neurosurgery, many studies on the clinical application of 3D printing technology, such as models for cranioplasty, patient-specific spinal instruments, and cerebral aneurysm models, have been reported^8–14^. However, the clinical implementation of a patient-specific 3D-printed brain tumor model system for daily surgical planning has not been established until now.

For brain tumor surgery, total resection of the tumor while preserving functional integrity should be considered a top priority. To achieve this goal, the selection of an optimal approach with stereoscopic understanding of related anatomical structures is the core issue. The current best practice in neurosurgical planning largely depends on the conceptual reconstruction of lesions in the surgeon’s head based on 2-dimensional magnetic resonance (MR) images or computer-aided image reconstruction incorporated into neuro-navigation, which is still not intuitive^15–19^. This kind of skill requires repeated practice and a high level of experience, which is difficult to achieve in the current training environment with limited opportunities^20^. For trainees, case lessons using a patient-specific 3D-printed disease model can be an effective tool to shorten the learning curve^19–21^. In addition, the complex nature of neurosurgical disease often makes it difficult for patients and caregivers without any expertise to understand about their situation. Explanations using the 3D-printed model may provide an intuitive understanding of the disease, surgical procedure, and risks^22,23^.

Currently, many medical device companies and medical startups around the world are providing services for producing clinical 3D-printed models in various fields, and their range and fields are gradually expanding^4^. It has been accepted that creating 3D-printed brain tumor models is more intricate than creating models of common cerebrovascular disease due to the anatomical complexity of the brain, diversity of lesions and adjunctive changes, difficulty in segmentation, and texture realization^24–27^. With the recent development of auto-segmentation and 3D-printed manufacturing technology, it has become practical to create a 3D-printed brain tumor model that meets all the requirements for clinical application^24–29^. In the field of neurosurgery, the applications of patient-specific 3D-printed brain disease models have also expanded, and several studies proved the effectiveness of these models for trainee education, patient education, surgical planning and simulation^24,26,28,29^. However, to apply and commercialize the 3D-printed brain tumor model for practical clinical use, the accuracy of a model that satisfies the neurosurgeon's fickle needs, a production periods tailored to the surgical schedule, and reasonable costs should be predetermined in a standardized and systematized manner. Here, we present our newly developed clinical application platform for patient-specific 3D-printed models of brain tumor patients and verification of its usefulness through a simulated clinical use.

## Methods

### Brain tumor segmentation

**Figure 1:**
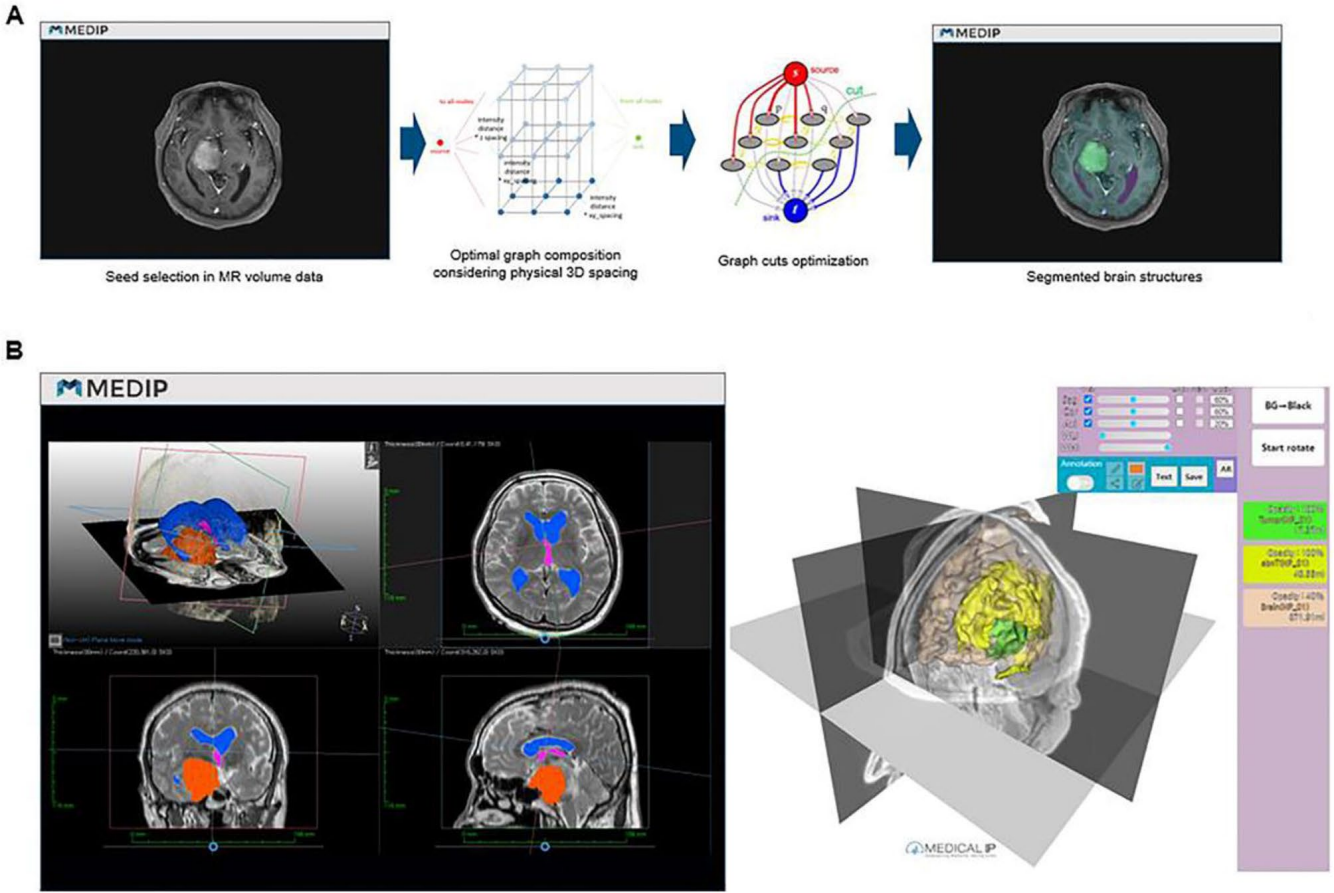
(**A**) Graph-cut algorithm. (**B**) Multiplanar reconstruction (MPR) function of MEDIP during the segmentation process. The finished result can be seen at the following link (http://medicalip.synology.me:8082/191011_NP01.html).

Segmentation is a term used in 3-dimensional modeling and refers to the extraction of regions that show the same signal in a specific image. The first step was to create software that can accurately segment the brain tumor and anatomical structures of the brain. In previous studies, manual drawing methods using threshold differences were unavoidable in some part of the segmentation process due to the complexity of the brain^25,28–30^. We automated most of the segmentation process using machine learning-based thresholds, region growing, and graph-cut algorithms and produced a program that can perform segmentation, reconstruction and rendering (MEDIP, http://medicalip.com/Medip, MEDICALIP, Seoul, Republic of Korea). When MR DICOM images of patients are registered in MEDIP, the preprocessing step removes the noise generated in the medical image before full-scale segmentation and is performed to improve image quality. Subsequently, when the user designates the region of interest (ROI), the specified ROI is replaced with seed information, and segmentation is performed for the area with the same seed information, such as the specified ROI, using the graph-cut algorithm (Fig. 1A), which is the core algorithm of MEDIP software. The graph-cut algorithm is an algorithm that separates the foreground from the background by configuring each pixel with graph intersections (nodes) and utilizing the difference in energy (flow network) between them. Segmentation is performed with the seed point specified by the user as the starting point. Using the methods of drawing a solid line with a pencil via an input device such as a mouse (Sketch method), creating a polygon by clicking on the coordinates and filling it inside (Polygon method) and filling the inside in a free form (Freedraw method), the user can directly specify the foreground and background. This technology was patented in the United States^31^. Since 3D medical images should be segmented within the actual distance (spacing) between each pixel and based on limited pixel and resolution information, optimization was carried out in the program. Segmentation is performed by the function Draw-cut on MEDIP, and semi-automated segmentation is carried out through this function. Using the Multi-Planar Reconstruction (MPR) function (Fig. 1B), the virtual 3D brain tumor model and the MR image can be overlapped while cross sectioning the desired reference point, so the segmentation can proceed while confirming that the process has been performed correctly. As a source image for segmentation, enhanced T1-weighted, T2-weighted, and T2 fluid-attenuated inversion recovery (FLAIR) MR images were used in combination. All MR image sequences tested in this study were performed using a 3.0-T GE Discovery MRI system (GE Healthcare), and the images were acquired with a 1.0-mm thick slabs and 1.0-mm spaces. The goal was to realize the brain tumor and the anatomical structures of the brain in 3 dimensions with the maximum utilization of the signal differences between the source image and the thin section image in 1-mm intervals.

### Virtual 3D model sharing and modification

The virtual 3D brain tumor model (virtual 3D model), which was primarily rendered after segmentation, can be shared with the requesting neurosurgeon and the 3D model manufacturer in an interactive way by sending the Uniform Resource Locator (URL) of the model uploaded to the web through mobile MEDIP (MODIP, MEDICALIP, Seoul, Republic of Korea). MODIP is a tool for real-time communication between the neurosurgeon and manufacturer, through which the prototype of the rendered virtual 3D model can be shared (example URL; http://147.47.229.147:8080/STLRendering/190513_brain.html). The neurosurgeon can check the result of the shared model and request modifications by entering text or drawing pictures using a smartphone or computer. MODIP is equipped with a function to rotate and resize the 3D model in all directions so that the accuracy of production can be checked in detail from multiple directions. By expressing the tumor and each anatomical structure in different colors, the anatomical structure can be clearly identified, and with customization functions such as annotation, color change, addition and subtraction of each structure, the neurosurgeon can describe or sketch the preferred modifications, add or remove the desired brain structure (blood vessels and nerves around the tumor, skull base structure) and change the color or transparency of the structure. If the tumor was located in the cranial base, a portion of the skull base structure adjacent to the tumor could be selected to tailor the model to only the desired range. After this interactive process, the neurosurgeon can finally confirm the model to proceed to the 3D printing step.

### Production of a patient-specific 3D-printed brain tumor model

**Figure 2:**
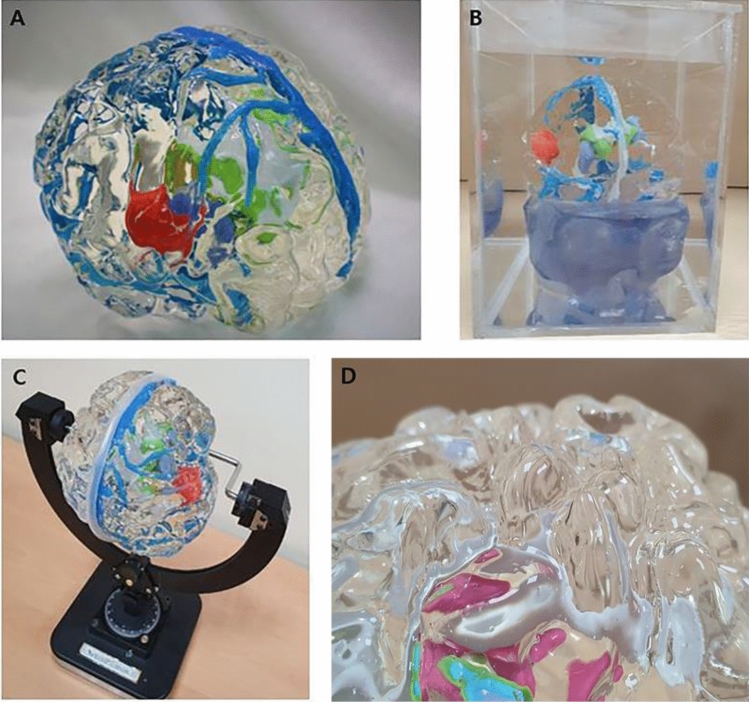
(**A**) The final 3D-printed brain model of right insular glioma. Normal brain parenchyma with expression of the gyri and sulci was reconstructed using transparent silicon material. The tumor is colored light red, the ventricle system is light gray, the caudate nucleus is yellow-green, the thalamus is light blue, and the major venous system is blue. The soft real brain-like texture of the model enables the neurosurgeon to simulate surgery by incising and excavating the area of interest. (**B**) The internal structure of the model can be clearly observed, and diffuse reflections can be eliminated by placing the model in the water tank. (**C**) The surgical approach can be fine-tuned with a dedicated fixator that can determine the rotation angle of the model in all directions. (**D**) The closure view of the model shows the details of gyri and sulci.

The production of the patient-specific 3D-printed brain tumor models consisted of three stages: creating a stereolithography (STL) file for 3D printing, printing physibles using a 3D printer, and performing a post-processing step, which includes manual editing. Authentic realization of the gyrus and sulcus using transparent silicon materials with a similar brain texture to see through intra-axial tumors is a key component of our model (Fig. 2A,D). The gyri and sulci-shaped moldings of the brain surface, brain tumor and associated brain structures are printed. After performing the subtraction process using the slicing program, if no overlap was observed among the brain parenchyma, tumor and other brain structures, these features were extracted into separate STL files and printed independently with the desired material and color. The brain parenchyma was sliced using the Flash Print program (FlashForge; No.518 XianYuan Road, Jinhua City, ZheJiang Province, China), and the gyri and sulci-shaped moldings were printed using acrylonitrile butadiene styrene (ABS) copolymers by the fused deposition modeling method (Guider2, [FlashForge; No.518 XianYuan Road, Jinhua City, ZheJiang Province, China]). The tumor and other associated structures were sliced using a Grab CAD program (Stratasys; 7665 Commerce Way Eden Prairie, MN 55344, USA) and printed with photovoltaic resin by the Polyjet method (J750 [Stratasys; 7665 Commerce Way Eden Prairie, MN 55344, USA]). The supports attached to the outputs were removed after printing, and the brain parenchyma was fumigated to smoothen the surface. After assembling the gyri and sulci-shaped moldings, outputted tumor and internal brain structures, the transparent silicone material with a similar texture to brain when hardened was injected into the molding and then and dried. After the silicone material was completely dried, the mold was removed, and the 3D-printed brain tumor model was finished by homogenizing the surface.

In addition, we developed assistive devices, including a rotatable water tank (Fig. 2B) to minimize diffuse reflection caused by the flexion of the gyri and sulci and a model fixator, which is similar to the head fixator used in brain surgery (Fig. 2C), to gain a better view of the internal structures and achieve precise surgical planning and simulation.

### Clinical usefulness validation

A simulated clinical validation was conducted so that neurosurgeons could check the clinical usefulness of a customized 3D-printed brain tumor model. A total of 10 cases of brain tumors with various pathologies, locations, and depths were retrospectively selected, and 3D-printed brain tumor models were produced (Table 1). The simulated clinical usefulness validation was executed at an allocation site of an exhibition booth during the 59th Annual Meeting of the Korean Neurosurgical Society (http://2019.kns-neurosurgery.or.kr) using an electronic questionnaire scenario with randomly chosen models for each volunteer (http://147.47.229.147:9090/brain/index.php). These processes were performed under institutional review board (IRB) approval from the 2 institutions (Seoul National University Hospital and Chungbuk National University Hospital) where the cases originated. The IRB of 2 institutions where the cases originated waived off the requirement for informed consent for the simulated clinical validation due to the no interaction with patients and the retrospective nature of the study. To assess the usefulness of the 3D printed brain tumor model in surgical planning for various tumor types and locations, the tumors were categorized according to the location depth: cortex, intermediate (outer tumor margin located less than 2 cm from the cortical surface), deep (outer tumor margin located between 2 and 4 cm from the cortical surface), and very deep (outer tumor margin located more than 4 cm from the cortical surface). A total of 32 neurosurgeons (14 faculty members, 11 fellows, and 7 residents) voluntarily participated for 2 days. The participants responded at a table separated by a partition and the surroundings were quiet. The process was carried out alone with the help of an assistant who presented MR images and 3D-printed brain tumor models.

**Table 1:**
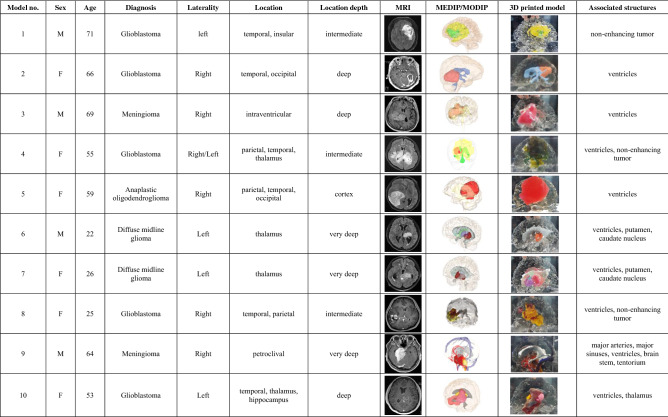
Clinical features of 10 fabricated 3D printed brain tumor models.

M, male; F, female; MRI, magnetic resonance image; 3D, 3-Dimensional

Questionnaires were administered about the decisions regarding surgical plans for a certain selected case based on MR images (enhanced T1, T2, T2 FLAIR sequences in the coronal, axial, sagittal planes) alone, a perspective 3D reconstructed brain tumor plan before printing (which can be rotated in all directions on a computer), and a 3D-printed brain tumor model (with the aid of a water tank and a fixator). The participants were repeatedly asked about the proper surgical position, including head rotation direction and degree, the location and extent of craniotomy, and the goal of the extent of tumor removal after inspecting the given MR images, perspective plan, and 3D-printed model. The surgical position questionnaire was answered by choosing objective item from supine, prone, and lateral. The choice of direction and degree of head rotation was determined by selecting the right/left side and then 0, 10, 30, 45, 60, 90, or 100 degrees. The area and extent of craniotomy were determined by drawing directly on the screen showing the skull image with the selected direction and degree of head rotation. To score the changes in degree of craniotomy size, we devised the following scoring system ranging from 0 to 3 points: 0 for less than a 25% change, 1 for a 25–50% change, 2 for a 0–75% change, and 3 for a 75–100% change in craniotomy size. Similarly, changes in craniotomy location were scored from 0 to 2 points: 0 for more than a 90% overlap, 1 for more than a 50% overlap, and 2 for less than a 50% overlap in craniotomy area. The participants were also asked if they would modify the goal of the surgery in terms of the extent of resection after inspecting the 3D brain tumor model. No time limit was set for determining the answers. An additional survey consisting of 5 questions was administered to evaluate the usefulness of the MR images and 3D-printed model in establishing surgical plans on a 5-point scale from 1 (not useful) to 5 (very useful). The statistical significance of the results was also examined according to respondent factors, such as their status and experience in brain tumor surgery, as well as tumor factors.

### Statistical analysis

The chi-square test, Fisher's exact test, two sample t-test and ANOVA were used to appropriately compare the results according to the abovementioned factors. All statistical analyses were performed using the R free statistical software package (version 3.4.0; http://www.r-proje ct.org/). A *p*-value less than 0.05 was considered statistically significant.

### Ethics declarations

The study was conducted in accordance with the Declaration of Helsinki and complies with the current laws of the countries in which it was performed. An independent ethics committee or institutional review board (Seoul National University Hospital, IRB No. 1811-040-986 and Chungbuk National University Hospital, IRB No. 2019-06-015-001) for each study site approved the study protocol and all procedures performed in this study were in accordance with the ethical standards as set. The institutional review boards waived off the requirement for informed consent for the simulated clinical validation due to the no interaction with patients and the retrospective nature of the study. However, participants those who appeared in the figures signed an informed consent to publish the images in an online open access publication.

## Results

### Usefulness of the 3D-printed brain tumor model

**Figure 3:**
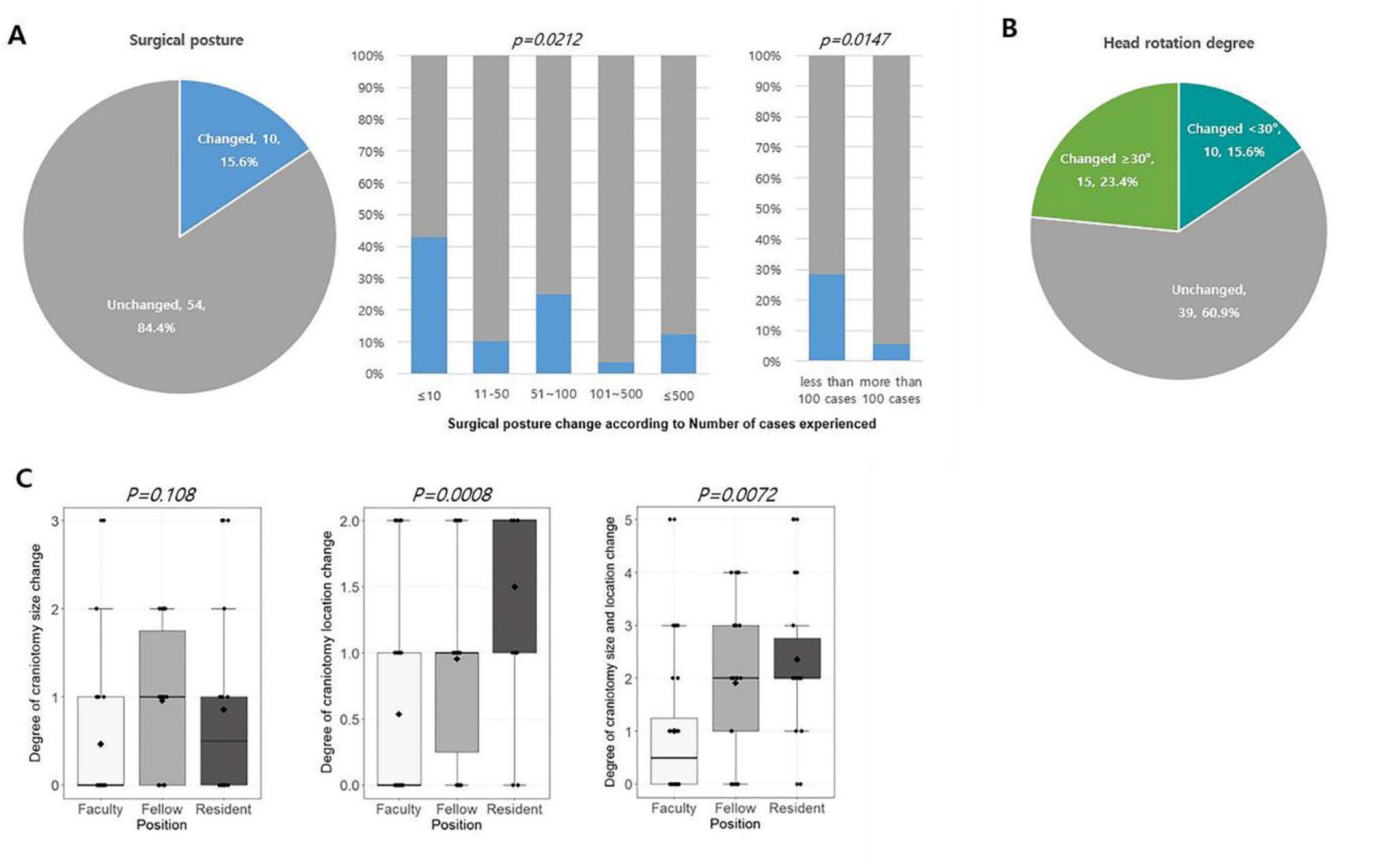
(**A**) Change in surgical posture from that determined from MR images after inspecting the 3D-printed brain tumor model. The surgical posture change rate shows significant differences among respondents according to the number of cases treated. (**B**) Change in head rotation degree from that determined from MR images after inspecting the 3D-printed brain tumor model. (**C**) The degree of change in craniotomy size and location was determined after inspecting the 3D -printed brain tumor model according to the training level of the respondents.

The simulated clinical usefulness validation acquired responses for 64 cases from 32 neurosurgeons, each of whom evaluated 2 out of the 10 prepared cases. In total, for 10 out of 64 cases (15.6%), the surgical posture determined by MR images only would be changed after inspecting the 3D-printed brain tumor model (Fig. 3A). These changes were significantly more common among respondents with less experience with brain tumor surgery (Fig. 3A and Supplementary Table 1). In particular, among 7 respondents who had experienced fewer than 10 surgical cases, 42.9% of the decisions were changed after inspecting the 3D-printed brain tumor model. Moreover, there was a significantly higher proportion (*p* = *0.0147*) of surgical posture changes in the group who had performed less than 100 cases of brain tumor surgery (8/28, 28.6%) than in the group who had performed more than 100 surgeries (2/36, 5.6%) (Fig. 3A and Supplementary Table 1). For a total of 25 out of 64 cases (39.1%), the degree of head rotation for the surgery was changed after inspecting the 3D brain tumor model (Fig. 3B), and these changes were not related to the respondents’ training level or experience (Supplementary Table 1). The changes in the location and extent of craniotomy size determined by MR images and the 3D-printed brain tumor model were also analyzed in a similar way. There were significant changes in craniotomy location only (*p* = *0.0008*) or both location and size (*p* = *0.0072)* after inspecting the 3D-printed brain tumor model among residents and/or fellows (Fig. 3C and Supplementary Table 1). The surgical plans made by faculty members were relatively consistent regardless of the information provided by either MR images or the 3D-printed brain tumor model. Tumor factors had little impact on the determination of the surgical positions or craniotomy design (Supplementary Table 2).

**Figure 4:**
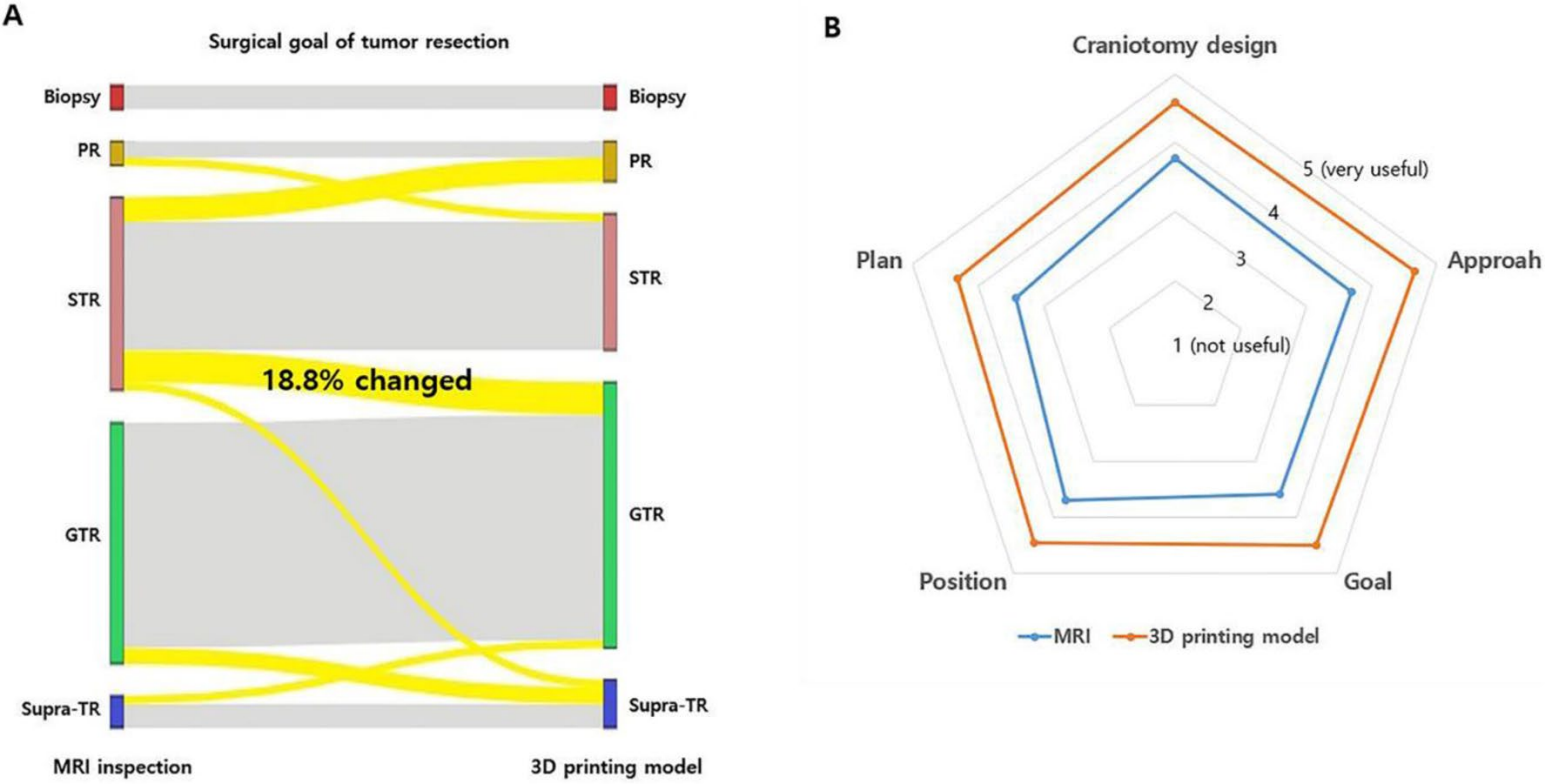
(**A**) Sankey diagram showing the change in the extent of tumor removal between the MRI-based and 3D-printed brain tumor model-based plans. Overall, for 12 out of 64 cases (18.8%), the surgical goal was modified after examining the 3D-printed brain tumor model. (PR, partial resection; STR, subtotal resection; GTR, gross total resection; Supra-TR, supra-total resection). (**B**) The result of the questions evaluating the usefulness of MR images and the 3D-printed model in establishing surgical plans, as scored on a 5-point scale from 1 (not useful) to 5 (very useful). In all questions, the 3D-printed brain tumor model scored higher than the MR images.

The surgical goals were modified in terms of the extent of resection after examination of the 3D-printed brain tumor model in 12 out of 64 cases (18.8%, Fig. 4A). Among them, the resection goal was lessened in 4 cases, while the resection extent was widened in 8 cases after inspecting the 3D-printed brain tumor model. Five questions, which were scored on a 5-point scale, on comparing MR images and the 3D-printed brain tumor model were asked; Q1. Usefulness in predicting and determining the location and extent of craniotomy (craniotomy design), Q2. Usefulness in determining the appropriate degree of head rotation (approach), Q3. Usefulness in determining the extent of tumor resection (goal), Q4. Usefulness in determining the surgical posture to remove the tumor effectively (position), and Q5. Usefulness in envisaging the entire process of the surgical resection (plan). The average scores showed that the 3D-printed brain tumor model was superior to MR images for surgical planning surgery in all aspects (Fig. 4B).

### Clinical application flow

**Figure 5:**
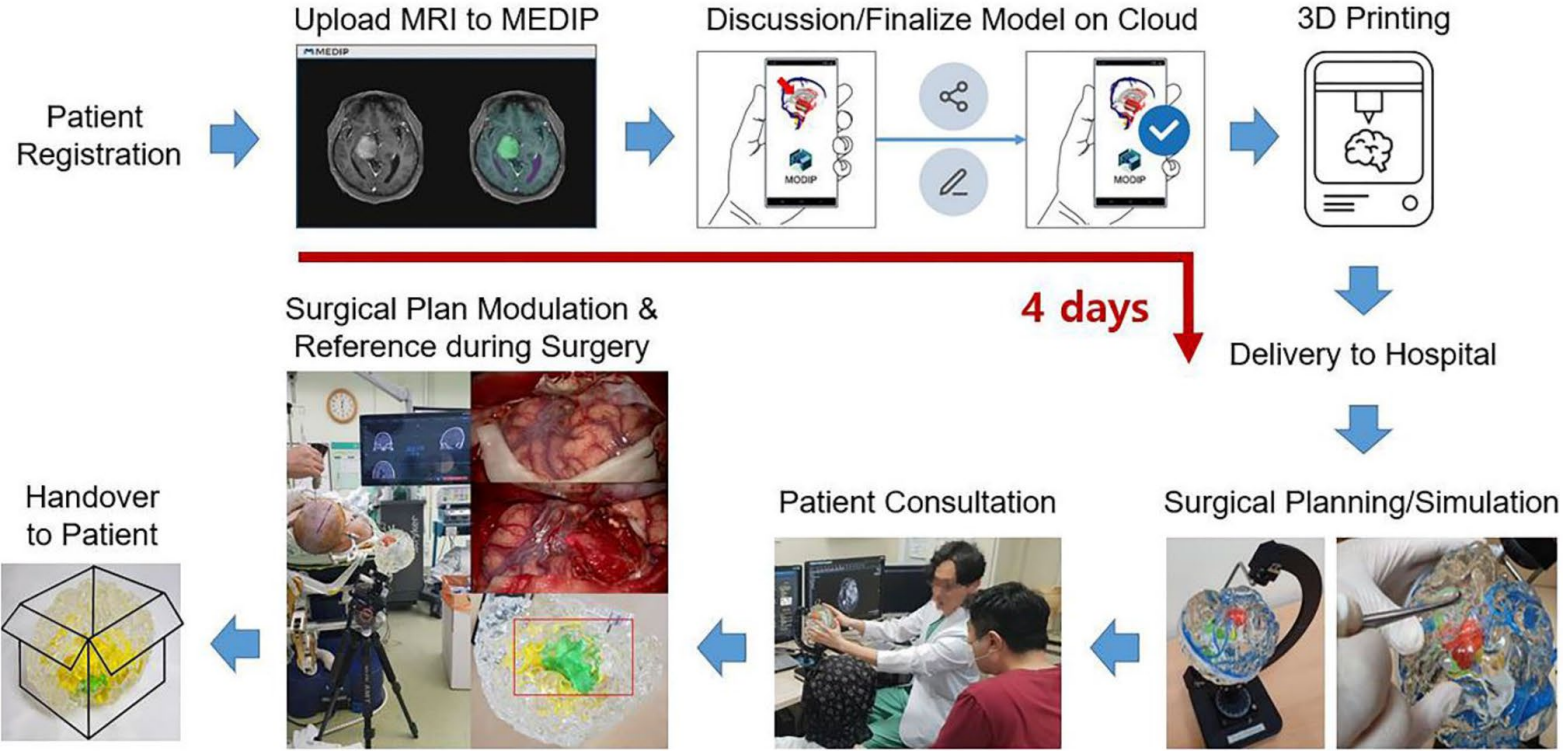
Clinical application flow of the 3D-printed brain tumor model.

We built a patient-specific 3D-printed brain tumor model production system from patient registration to delivery of the final model to the hospital, and this process was completed within 4 days (Fig. 5). This is possible thanks to the interactive online system for making perspective 3D models and standardized 3D-printed manufacturing processes. If there is a candidate who needs a 3D-printed brain tumor model produced for surgical planning, a neurosurgeon uploads the DICOM files of the MR images to the MEDIP after obtaining consent from the patient. The segmentation process is followed, and the result is transferred to a cloud-based system using the MODIP interface in approximately one day. The perspective 3D model is finalized after online discussion and fine tuning between the neurosurgeons and technicians during the following day. Then, the customized 3D printed brain tumor model is produced, and the finished product is delivered in 2 days.

Using a patient-specific 3D-printed brain tumor model, neurosurgeons can make a surgical plan, simulate a surgery, and use the model to explain the surgery to the patient (Fig. 5). Neurosurgeons can obtain insights into the stereoscopic location of the tumor, appropriate surgical corridor or approach to the tumor, effective craniotomy design, and goal of the surgery using the 3D-printed brain tumor model. The transparent and soft nature of the model with exquisite realization of the gyri and sulci provides intuitive ideas for surgical planning. However, diffuse reflection may limit the view of internal structures due to the transparent flexion of the gyri and sulci, especially when a deep-seated tumor is encountered. To compensate for this diffuse reflection, a rotatable water tank was devised to clearly see through the internal structures by placing the model in the water. In addition, more sophisticated surgical planning can be enabled by the model fixator, which can simulate the surgical position by replicating the angle of head rotation in all directions. By incising and excavating the delicately implemented gyri and sulci having similar physical properties to the brain, neurosurgeons can determine the effective cortical window to the tumor, avoiding injury to eloquent areas and critical structures. The model can also be used for patient consultations before surgery so that patients or caregivers can gain a better understanding of the nature of the disease and surgery.

During surgery, the 3D-printed model can be installed in the operating room according to the planned surgical position and can be used as a reference for surgical plan modulation and surgical anatomy during surgery (Fig. 5). After the surgery, the 3D-printed model is retrieved and provided to the patient upon request.

## Discussion

Since Spottiswoode et al. reported the first 3D-printed brain tumor model, there have been continuous efforts in developing 3D-printed brain tumor models^25^. These pilot models were created for training and education purposes, surgical simulation, and surgical planning^20,24,25,28–30,32,33^. Tumors in various locations, including the cerebrum, cerebellum, brain stem and pituitary tumors, have thus far been implemented^20,24,28,29,33^. Most of the early models focused on the brain tumor itself without paying much attention to the surrounding brain structures, which provides insufficient information to create a delicate surgical plan. Although recent models gave shape to peritumoral neurovascular structures, there are still limitations to designing a surgical plan unless the whole brain is embodied, especially for intra-axial tumors.

To further progress from the previous phase of the 3D-printed model within academic boundaries, we have established a full-cycle platform for the clinical application of a 3D-printed brain tumor model system. Although many efforts have been exerted to introduce 3D-printed brain tumor models to the clinical field, previous studies have only suggested a possibility for clinical entry or limited educational purposes^24–26,28,29^. To overcome the hurdles of the practical application of 3D-printed brain tumor models in daily clinical use, we have standardized the manufacturing process, shortened the production period, advanced the design and materials, devised auxiliary equipment, and reduced the manufacturing cost. These points are the differences and superiorities of our platform from those of other previously reported models. Implementing precise segmentation in a short time with an interactive sharing system for the perspective 3D model was the most strenuous challenge in this study. When the neurosurgeon registers DICOM files to MEDIP, subsequent steps, including image preprocessing for quality improvement, overall configuration of the graphs based on foreground/background seed settings, and semiautomated graph-cut segmentation, are executed sequentially without delay. The purpose of the MODIP platform is to enable the neurosurgeon to check the rendered perspective 3D model immediately on any kind of device without installing specific applications and communicate with multiple users. This interactive tool contributes to effective discussions for developing and modifying models in a short time.

The top priority of 3D-printed models for surgical planning is to mimic all anatomical structures as realistically as possible. Previously published 3D brain tumor models had mainly focused on the tumor itself due to issues with cost or technical problems and based on the purpose of the study^24,26,28,29^. We thought that implementing a 3D brain tumor model with the whole brain parenchyma shown in transparent soft material with precise expression of the gyri and sulcus is important for use in surgical simulation. This is also the differentiated advantages of our models. To this end, we discovered a silicon material that is transparent and has similar properties to the brain when hardened. On the other hand, it was also not easy to model a gyri and sulci similar to the real brain. Printing the gyri and sulci similar to the brain using conventional 3D printer properties elicited a problem: the shape collapsed during printing due to the weight of the product. Through trial and error, we established a process to make external moldings and inject transparent silicone material into these moldings. We found that this process not only facilitates the expression of delicate real brain-like gyri and sulci but also reduces the production time and cost. Another effort to reduce the production time of the 3D printing process was the application of a polyjet (photopolymer + jetting) printer that sprays materials and solidifies them. When we used a fused deposition modeling (FDM)-type 3D printer that can only print with a single material with a single nozzle, the production time was much longer due to postprocessing work, which included removing the supports, surface-treating and assembling for the individual structures. However, the polyjet printer allows for simultaneous printing with materials of multiple properties, thus producing the model in the assembled form. Furthermore, support removal and surface treatment can be performed by the water-jet equipment, which reduces the production time and improves precision. Although we showed only 10 representative cases of brain tumor models in with various pathologies, locations, and depth in this study, there are no limitations on anatomical location of interest or age of patients to produce the model. Even very small and delicate structures such as blood vessels and cranial nerves can be implemented as soon as they are visible on MRI.

In the simulated clinical validation executed in this study, we confirmed that the 3D-printed brain tumor model is more helpful for neurosurgeons with less experience in planning surgery. The changes made to the surgical plans after inspecting the 3D-printed brain tumor models were crucial in terms of patient safety. With the aid of novel tools such as 3D-printed brain tumor models, novice neurosurgeons can perform surgery confidently with intuitive information about the tumor, and the learning curve is expected to be shortened. The additional comments from the participants in the simulated clinical validation revealed that the 3D-printed brain tumor model helped determine the surgical plan by providing both microscopic views of the tumor as well as views from multiple directions after the model was fixed and the brain parenchymal portion was dissected out; this provided insight into the depth of the tumor location. Additionally, the model could help reduce the craniotomy size and increase the accuracy of the craniotomy site. The participants also mentioned that the 3D-printed model has merits in transferring tumor information among physicians engaged in patient management.

For neurosurgeons, the 3D-printed brain tumor model will enable an accurate craniotomy, ensure the maximum level of safety during resection, and minimize neurological deficits caused by surgery, thus, a safe surgery can be performed within the appropriate range and time. This will eventually result in a lower infection rate, rapid recovery, and a decrease in the incidence of neurological deficits in patients. Using the model to explain the surgical procedure could improve the patient’s psychological status and rapport with the doctor by enhancing their surgical understanding. In addition, sharing the 3D-printed brain tumor model with surgical assistants, nurses, anesthesiologists, and intensive care unit physicians can improve the quality of care for patients by enhancing the understanding of the surgery throughout the surgical team. This ultimately leads to benefits for the patients.

## Conclusion

We established a 3D-printed brain tumor model production system that is ready to use in daily clinical practice for neurosurgery. The effectiveness of this system was tested in clinical field and could be confirmed by simulated clinical validation. We hope that this system will be widely introduced to the neurosurgery clinic as a new gear for the development of the next step for the future surgery.

## Supplementary Information

Supplementary Table 1.

Supplementary Table 2.
