# Supplementary material for: Clinical application of patient-specific 3D printing brain tumor model production system for neurosurgery
Source: Sci Rep. 2021 Mar 26;11:7005. doi: 10.1038/s41598-021-86546-y (PMC7998007; doi:10.1038/s41598-021-86546-y)
Supplement: Supplementary file 1 — Supplementary Table 1. [file 41598_2021_86546_MOESM1_ESM.docx]

**Supplementary table 1.** Changes of surgical planning according to the respondent variables

| **Respondent class** | | | **Surgical posture changes** | | | **Changes in degree of head rotation of surgical position** | | | | **changes in craniotomy size** | | **changes in craniotomy location** | | **changes in craniotomy size and location** | |
| --- | --- | --- | --- | --- | --- | --- | --- | --- | --- | --- | --- | --- | --- | --- | --- |
| **Group** | **Class** | **No. of respondents** | **yes** | **no** | **p-value** | **No change** | **<30°** | **≥30° or direction change** | **p-value** | **score** | **p-value** | **score** | **p-value** | **score** | **p-value** |
| Position | Faculty | 14 | 2 | 26 | *0.1487* | 19 | 5 | 4 | *0.5063* | 0.46±0.74 | *0.108* | 0.54±0.74 | *0.0008** | 1.00±1.33 | *0.0072** |
|  | Fellow | 11 | 6 | 16 |  | 13 | 2 | 7 |  | 0.95±0.79 |  | 0.95±0.72 |  | 1.91±1.38 |  |
|  | Resident | 7 | 2 | 12 |  | 7 | 3 | 4 |  | 0.86±1.10 |  | 1.50±0.76 |  | 2.36±1.45 |  |
| Training | Faculty | 14 | 2 | 26 | *0.0993* | 19 | 5 | 4 | *0.3121* | 0.46±0.74 | *0.032** | 0.54±0.74 | *0.0017** | 1.00±1.33 | *0.0026** |
|  | Fellow and Resident | 18 | 8 | 28 |  | 20 | 5 | 11 |  | 0.92±0.91 |  | 1.17±0.77 |  | 2.08±1.4 |  |
| Number of cases experienced (brain tumor surgery cases) | ≤10 | 7 | 6 | 8 | *0.0212** | 7 | 1 | 6 | *0.2554* | 0.71±0.91 | *0.999* | 1.07±0.83 | *0.2434* | 1.79±1.42 | *0.4192* |
|  | 11-50 | 5 | 1 | 9 |  | 5 | 3 | 2 |  | 0.9±0.99 |  | 1.7±0.88 |  | 2±1.63 |  |
|  | 51~100 | 2 | 1 | 3 |  | 2 | 0 | 2 |  | 0.75±0.5 |  | 1.25±0.5 |  | 2±0.82 |  |
|  | 101~500 | 14 | 1 | 27 |  | 21 | 4 | 3 |  | 0.75±0.93 |  | 0.82±0.82 |  | 1.57±1.57 |  |
|  | ≤500 | 4 | 1 | 7 |  | 4 | 2 | 2 |  | 0.38±0.52 |  | 0.38±0.74 |  | 0.75±1.04 |  |
| Number of cases experienced (100 brain tumor surgery cases) | less than 100 cases | 14 | 8 | 20 | *0.0147** | 14 | 4 | 10 | *0.1203* | 0.79±0.88 | *0.588* | 1.11±0.79 | *0.061* | 1.89±1.4 | *0.174* |
|  | more than 100 cases | 18 | 2 | 34 |  | 25 | 6 | 5 |  | 0.67±0.86 |  | 0.72±0.81 |  | 1.39±1.5 |  |

***statistically significant
