# Supplementary material for: Clinical application of patient-specific 3D printing brain tumor model production system for neurosurgery
Source: Sci Rep. 2021 Mar 26;11:7005. doi: 10.1038/s41598-021-86546-y (PMC7998007; doi:10.1038/s41598-021-86546-y)
Supplement: Supplementary file 2 — Supplementary Table 2. [file 41598_2021_86546_MOESM2_ESM.docx]

**Supplementary table 2.** Changes of surgical planning according to the tumor factors

| **Tumor factors** | | | **Surgical posture changes** | | | **Changes in degree of head rotation of surgical position** | | | | **changes in craniotomy size** | | **changes in craniotomy location** | | **changes in craniotomy size and location** | |
| --- | --- | --- | --- | --- | --- | --- | --- | --- | --- | --- | --- | --- | --- | --- | --- |
| **Group** | **Class** | **No. of cases** | **yes** | **no** | **p-value** | **No change** | **<30°** | **≥30° or direction change** | **p-value** | **score** | **p-value** | **score** | **p-value** | **score** | **p-value** |
| Depth of tumor location | cortex | 1 | 2 | 4 | *0.6156* | 3 | 0 | 3 | *0.6826* | 1.67±1.21 | *0.244* | 1.17±0.75 | *0.5494* | 2.83±1.94 | *0.3884* |
|  | intermediate | 3 | 3 | 22 |  | 17 | 4 | 4 |  | 1.08±0.91 |  | 1.32±0.75 |  | 2.4±1.22 |  |
|  | deep | 3 | 3 | 19 |  | 13 | 4 | 5 |  | 1.64±1 |  | 1.5±0.86 |  | 3.14±1.73 |  |
|  | very deep | 3 | 2 | 9 |  | 6 | 2 | 3 |  | 1.36±1.03 |  | 1.64±0.67 |  | 3±1.34 |  |
| Tumor type | intra-axial | 8 | 8 | 43 | *1.0* | 31 | 8 | 2 | *0.9988* | 1.39±0.98 | *0.788* | 1.43±0.76 | *0.8474* | 2.82±1.47 | *0.8019* |
|  | extra-axial | 2 | 2 | 11 |  | 8 | 2 | 3 |  | 1.31±1.11 |  | 1.38±0.87 |  | 2.69±1.7 |  |
